# Characterization of extended-spectrum cephalosporins and fluoroquinolone resistance of a *Salmonella enterica* serovar Thompson isolate from ready-to-eat pork product in China

**DOI:** 10.3389/fmicb.2022.964009

**Published:** 2022-09-15

**Authors:** Lili Li, Rikke Heidemann Olsen, Jian Xiao, Meidan Liang, Hecheng Meng, Shifu Peng

**Affiliations:** ^1^Institute of Food Safety and Nutrition, Jinan University, Guangzhou, China; ^2^School of Food Science and Engineering, South China University of Technology, Guangzhou, China; ^3^Department of Veterinary and Animal Sciences, Faculty of Health and Medical Sciences, University of Copenhagen, Frederiksberg C, Denmark; ^4^Guangzhou Food Inspection Institute, Guangzhou, China; ^5^Department of Environment and Health, Jiangsu Center for Disease Control and Prevention, Nanjing, China

**Keywords:** *Salmonella* Thompson, *bla*_CMY−2_, *qnrS1*, *qepA8*, ready-to-eat pork product

## Abstract

*Salmonella* is a leading cause of foodborne illness worldwide and is a common concern in food safety. *Salmonella enterica* displaying resistance to extended-spectrum cephalosporins (ESCs) and fluoroquinolone (FQs) has been deemed a high-priority pathogen by the World Health Organization. Co-resistance to ESCs and FQs has been reported in *S. enterica* serovar Thompson (*S*. Thompson). However, the genetic context of ESCs and FQs resistance genes in *S*. Thompson lacks sufficient characterization. In this study, we characterized a multi-drug resistant (MDR) *S*. Thompson isolate recovered from a retail ready-to-eat (RTE) pork product in China. Short- and long-read sequencing (HiSeq and MinION) of the genome identified the presence of *bla*_CMY−2_, *qnrS1*, and *qepA8*, along with 11 additional acquired antimicrobial resistance genes, residing on a 152,940 bp IncA/C plasmid. Specifically, the *bla*_CMY−2_, *qnrS1*, and *qepA8* genes were located in insertion sequences (ISs) and integron mediated mobile genetic structure, *sugE*-*blc*-*bla*_CMY−2_-IS*Ec9*, IS*26*-*orf6*-*qnrS1*-*orf5*-IS*Kpn19*, and *intl1*-*qepA8*-*orf10*-IS*91*-*orf1*-*dfrA12*-*orf11*-*aadA2*-*qacE*Δ*1*-*sul1*, respectively. Each gene was identified in various bacteria species, indicating their high transfer ability. The plasmid was found to be transferable to *Escherichia coli* J53 by conjugation and resulted in the acquiring of multiple resistances in the transconjugants. The plasmid is closely related to plasmids from two human *S*. Thompson strains isolated in different regions and years in China. Moreover, core-genome Multi Locus Sequence Typing (cgMLST) and phylogenetic analysis based on global 1,868 *S*. Saintpaul isolates showed that the *S*. Thompson isolate was highly epidemiologically linked to a human isolate in China. Our findings suggest that Chinese RTE pork products are a possible source of human pathogenic ESCs and FQs co-resistant *S*. Thompson. Furthermore, the results underline the important role of conjugative plasmids in acquiring and transmission of ESCs and FQs resistance in *S*. Thompson isolates, which need continuous investigation.

## Introduction

*Salmonella* is a leading cause of food-borne illness worldwide (Chen et al., 2019). There are over 2,600 serovars of *Salmonella*. Among them, *S. enterica* subsp. *enterica* serovar Thompson (*S*. Thompson) is one of the most frequent *Salmonella* serovars in humans and is commonly associated with poultry (Shah et al., 2017; Eun et al., 2019; Qi et al., 2019; Zhou et al., 2019). *S*. Thompson contaminated meat products have been reported as frequent causes of human salmonellosis (Gaulin et al., 2017; Suijkerbuijk et al., 2017; Marder et al., 2018; Eun et al., 2019). Antimicrobial therapy (e.g., ciprofloxacin in adults and ceftriaxone in children) can be lifesaving in these patients (Crump et al., 2015). There is increasing concern over the emergence and increased incidence of multi-drug resistant (MDR) *Salmonella* strains, especially those resistant to extended-spectrum cephalosporins (ESCs) and fluoroquinolones (FQs), which have been deemed a high-priority pathogen by the World Health Organization (Collignon et al., 2016).

Cephalosporins resistance is mediated predominantly by extended-spectrum β-lactamases (ESBLs), AmpC β-lactamases, and carbapenemase (Arlet et al., 2006). Various β-lactamases, including *bla*_CTX−M_, *bla*_DHA−1_, and *bla*_SHV_, have been reported in *S*. Thompson isolates (Zhou et al., 2019; Elbediwi et al., 2021). Reduced susceptibility to FQs is associated with chromosomal mutations and acquisition of AMR genes, such as efflux pumps encoding genes and plasmid-mediated quinolone resistance (PMQR) genes (*qnr, aac(6*′*)-Ib-cr, oqxAB*, and *qepA*) (Cuypers et al., 2018). Co-occurrence of ESCs and FQs resistance genes has so far only been reported in a *S*. Thompson isolate from Chicken in China (Zhou et al., 2019). The co-existence and co-transfer of ESCs and FQs resistance genes in *Salmonella* may seriously compromise treatment options, especially for invasive salmonellosis (Crump et al., 2015). However, the genetic context and transferability of ESCs resistance and PMQR genes have not been thoroughly investigated in *S*. Thompson.

The aim of this study was to characterize the genetic context of ESCs and FQs resistance genes in a *S*. Thompson isolate recovered from a ready-to-eat (RTE) pork product in Guangzhou, China, and to analyze its possible origin as well as transferability, in order to gain insight into their public health impact.

## Materials and methods

### Strains isolation and identification

During our routine surveillance of foodborne pathogens on various food products, a *Salmonella* isolates (named GSJ/2017-*Sal.-*009, hereafter 17Sal009) was recovered from a retail RTE dumpling with pork and cabbage stuffing in Guangzhou, Southern China, in 2017. The isolate was identified by biochemical confirmation using API 20E test identification test strips (bioMérieux, France), as well as amplification of the *invA* gene by PCR (Bai et al., 2016). The serovar was determined by the slide agglutination test, using *Salmonella* antisera (SSI Diagnostica, Denmark) according to the Kauffmann–White scheme.

*E. coli* ATCC 25922 and *E. coli* J53 (sodium azide resistant) were used as the quality control for antimicrobial susceptibility testing and recipient strain for conjugation, respectively. All the strain was routinely grown for 12–24 h at 37°C on either Luria–Bertani (LB) broth or LB agar (Guangdong Huankai Microbial Sci &Tech, Guangzhou, China).

### Antimicrobial susceptibility testing

Susceptibility of *S*. Thompson 17Sal009 to a panel of antimicrobial drugs (Hangzhou Microbial Reagent Co., Ltd., China), including amikacin (30 μg), ampicillin (10 μg), amoxicillin clavulanic acid (20/10 μg), ampicillin-sulbactam sodium (10/10 μg), azithromycin (15 μg), aztreonam (30 μg), cefazolin (30 μg) (1st generation), cefoxitin (30 μg) (2nd generation), cefuroxime (30 μg) (2nd generation), cefotaxime (30 μg) (3rd generation), ceftazidime (30 μg) (3rd generation), cefepime (30 μg) (4th generation), chloramphenicol (30 μg), ciprofloxacin (5 μg), doxycycline (30 μg), ertapenem (10 μg), fosfomycin (200 μg), gentamicin (10 μg), imipenem (10 μg), meropenem (10 μg), netilmicin (30 μg), piperacillin (100 μg), streptomycin (10 μg), tigecycline (15 μg), tetracycline (30 μg), tobramycin (10 μg), and trimethoprim/sulfamethoxazole (23.75/1.25 μg) (Hangzhou Microbial Reagent Co., Ltd., China), were determined by disk diffusion antimicrobial susceptibility testing (Clinical Laboratory Standards Institute (CLSI)., 2018). Production of ESBL was confirmed by the disk diffusion clavulanate inhibition test using ceftazidime and cefotaxime and an ESBL producing *S*. *typhimurium* isolates 17Sal008 was used as a positive control [Li et al., 2022; Clinical Laboratory Standards Institute (CLSI)., 2018]. Minimum inhibitory concentrations (MICs) of 17Sal009, *E. coli* J53, and transconjugants to ciprofloxacin, nalidixic acid, and cefotaxime (Sigma-Aldrich, St. Louis, MO) were determined by broth microdilution (Clinical Laboratory Standards Institute (CLSI)., 2018). The final concentrations of antibiotics used were 0, 0.0075, 0.015, 0.03, 0.06, 0.12, 0.24, 0.5, 1, 2, and 4 μg/ml for ciprofloxacin, 0, 0.125, 0.25, 0.5, 1, 2, 4, 8, 16, 32, and 64 μg/ml for nalidixic acid and 0, 0.5, 1, 2, 4, 8, 16, 32, 64, 128, and 256 μg/ml for cefotaxime. Results were interpreted according to the CLSI breakpoints [Clinical and Laboratory Standards Institute (CLSI), 2018]. For ciprofloxacin, isolates with MICs ≤0.06 μg/ml were considered susceptible, while those with MICs of ≥ 1 μg/ml were considered resistant. For nalidixic acid, isolates with MICs ≤16 μg/ml were considered susceptible, while those with MICs of ≥32 μg/ml were considered resistant. For cefotaxime, *Salmonella* isolates with MICs of ≤1 μg/ml were considered susceptible, and those with MICs ≥4 μg/ml were categorized as resistant. Reference strain *E. coli* ATCC 25922 served as quality control. Diameters of disks were presented as mean values from replications with standard errors. All measurements were performed in duplicates and each experiment was repeated three times. MDR referred to non-typhoidal *Salmonella* isolates that were resistant to ampicillin, chloramphenicol, and trimethoprim/sulfamethoxazole (Gordon et al., 2008).

### Whole-genome sequencing and annotation

Genomic DNA of isolate 17Sal009 was extracted using a commercial DNA extraction kit (Magen, Guangzhou, China) following the manufacturer's recommendations. The whole genome of the isolate was sequenced on Illumina Hiseq × 10 with 150 bp paired-end reads (MajorBio Co., Shanghai, China) and MinION (Oxford Nanopore, Oxford, United Kingdom). For the Illumina platform, Initial data quality inspection was performed with FastQC (v0.11.9, https://www.bioinformatics.babraham.ac.uk./projects/fastqc) and then reads were filtered and trimmed using Cutadapt (v1.17) to discard the low-quality reads that contained ambiguous nucleotides or a quality score lower than 20 (Martin, 2011). For the MinION platform, the library was prepared using the ONT 1D ligation sequencing kit (SQK-LSK109) with the native barcoding expansion kit (EXP-NBD104). Fast5 files were basecalled using Guppy (v3.15) and output DNA sequence reads with Q > 7 were saved as fastq files. We assessed read statistics including quality scores and read lengths using NanoStat (v1.1.2, https://github.com/wdecoster/nanostat). The genome was assembled using a combination of short- and long-reads by SPAdes V3.14.0 (Bankevich et al., 2012) and Unicycler hybrid assembler V0.4.8 (Wick et al., 2017), and annotated by Prokka V1.14.6 (Seemann, 2014).

Clonal analysis was assessed by MLST 2.0 (https://cge.food.dtu.dk/services/MLST/). PlasmidFinder V2.1 was used to identify plasmid replicon types (Carattoli and Hasman, 2020). The presence of acquired antimicrobial resistance genes and mutations in the quinolone resistance-determining regions (QRDR) (*gyrA, gyrB, parC*, and *parE*) was assessed by ResFinder V4.1 (Bortolaia et al., 2020), and were further confirmed (based on 100% sequence coverage and ≥99% nucleotide identity) by BLASTn against nr database with default parameters (http://blast.ncbi.nlm.nih.gov/Blast.cgi). Virulence factors were predicted by the Virulence Factor Database (VFBD; http://www.mgc.ac.cn/VFs/ (accessed on 30 Jul 2022) (Chen, 2004). The complete plasmid sequence was BLASTn against the nr database with default parameters. The sequences showed 100% coverage and ≥99% nucleotide identity, as well as selected plasmids sharing highly similar backbone, were selected for comparison. The map of plasmid comparison was generated by BRIG 0.95-dev.0004 (Alikhan et al., 2011).

### Phylogenetic analysis of the genomic sequences

In order to assess the relatedness of 17Sal009 with other *S*. Thompson strains from different sources and countries, we retrieved all 1,868 genome sequences of *S*. Thompson that have been released from EnteroBase databases and performed core-genome Multi Locus Sequence Typing (cgMLST) (cgMLST scheme available on EnteroBase, https://enterobase.warwick.ac.uk, accessed on 18 Oct 2021) (Supplementary Table S1). Similar but non-identical strains [strains showing different core genome Sequence Types (cgST)] were identified in EnteroBase by using the hierarchical clustering method (HierCC) that allows for the grouping of strains into hierarchical clusters (HCs) that can differ up to a specified and fixed number of cgMLST alleles. This number is indicated by the suffix following “HC” (e.g., HC5 for 5 cgMLST allelic differences). Isolates belonging to the same HC10 cluster were considered possible epidemiologically linked, and isolates belonging to the same HC5 cluster were considered highly probably epidemiological linked (Bonifait et al., 2021). A minimum-spanning tree was created from cgMLST allelic differences in EnteroBase using GrapeTree with the RapidNJ algorithm (Zhou et al., 2019).

### Conjugation

The transferability of the plasmid was assessed by performing the conjugation experiment, using solid mating on a filter (Whatman, Maidstone, UK), in which the sodium azide-resistant E. coil strain J53 was used as a recipient strain (Hammerum et al., 2016). Briefly, recipient and donor strains were inoculated into LB broth and cultured overnight at 37°C. The next day, cells were harvested, washed with saline, mixed together in a ratio of 1:1, and spotted on to 0.45 μm-pore-size filter (Millipore) on LB plates. They were also spotted individually on LB plates as controls. After overnight incubation at 37°C, mating spots were washed and resuspended in saline; and different dilutions were plated on LB media containing 150 μg/ml sodium azide and 4, 8, or 16 μg/ml of cefotaxime to select transconjugants. Control spots were transferred to the same selective media to make sure that no growth was observed.

The conjugation frequency was calculated as the ratio of transconjugants over the number of recipients. The transfer of the plasmid was confirmed by PCR targeting the blaCMY-2 gene with primer CMYF (5′- CTCGACACGGACAGGGTTAG−3′) and CMYR (5′- TATTCCGGGTATGGCCGTTG−3′), as well as the E. coli uidA household gene with the primers UIDF (5′-TGGAATTTCGCCGATTTTGC-3′) and UIDF (5′-ATTGTTTGCCTCCCTGCTGC-3′) (Heijnen and Medema, 2016). The plasmid DNA was extracted from a selected transconjugant by a commercial plasmid extraction kit (Magen, Guangzhou, China) following the manufacturer's recommendations, and further sequenced on the Illumina Hiseq platform (MajorBio Co., Shanghai, China).

### Nucleotide sequence accession number

The assembly genome sequences of *S*. Thompson and pSal009 were deposited in GenBank under the accession number: CP050833.1, CP050832. The raw Illumina sequence data were deposited in the Enterobase database under the barcode numbers: SAL_JB2919AA.

## Results

### Identification of *Salmonella*

The isolate was confirmed as *S. enterica* serovar Thompson, serotype 6,7:r:1,5 by biochemical confirmation, 16S rRNA gene sequencing, and serotyping.

### Antimicrobial susceptibility

Disk diffusion antimicrobial susceptibility testing showed the isolate was resistant to ampicillin, amoxicillin-clavulanic acid, chloramphenicol, cefazolin, cefotaxime, ceftazidime, tetracycline, doxycycline, and trimethoprim/sulfamethoxazole, intermediate resistant to ciprofloxacin, aztreonam, cefoxitin, and cefuroxime, and was susceptible to gentamicin, tigecycline, fosfomycin, tobramycin, amikacin, piperacillin, ertapenem, imipenem, meropenem, netilmicin, cefepime, ampicillin-sulbactam sodium and produce ESBL. The isolate exhibited MIC values of ciprofloxacin, nalidixic acid, and cefotaxime for 2, 32, and 128 mg/L, respectively.

### General features of the *S*. Thompson 17Sal009 genome

The complete genome sequence of *S*. Thompson 17Sal009 contained a circular 4,897,518 bp chromosome with the G+C content of 52.3%, and a plasmid denoted as pSal009. There were 4,875 predicated CDs in the whole genome sequence, including 107 RNA genes. Multi-locus sequence typing analysis showed that 17Sal009 belongs to sequence type 26 (ST26).

**Figure 1:**
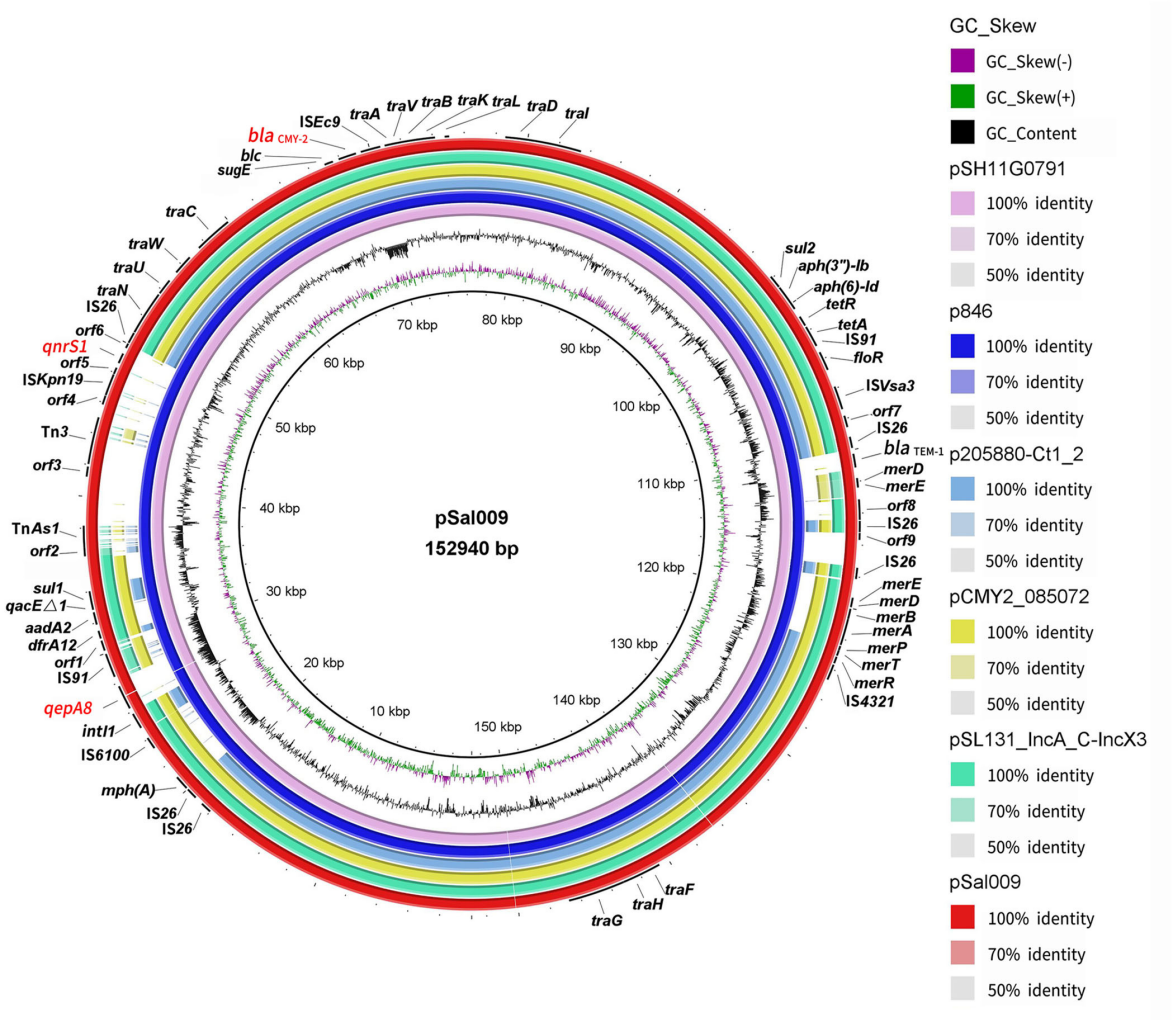
Sequence comparison of plasmid pSal009 identified in *S*. Thompson 17Sal009 with closely related plasmid pSH11G0791 and p846 as well as similar plasmids (p205880-Ct1/2, pCMY2_085072, and pSL131_IncA/C-IncX3) sharing similar backbone in different bacteria species in BRIG. GC content and GC skew of pSal009 are depicted in the inner rings. The genes located on pSal009 are annotated at the outside black ring. The *bla*_CMY−2_, *qnrS1*, and *qepA8* genes are marked red.

A total of 14 acquired antimicrobial resistance genes were identified by ResFinder, which encoded resistance to eight different antimicrobial classes, including cephalosporins, fluoroquinolones, phenicol, aminoglycoside, sulphonamide, trimethoprim, macrolide, and tetracycline. Mutations were not identified in the quinolone resistance-determining regions (*gyrA, gyrB, parC*, and *parE*) in 17Sal009. All 14 genes were located on the plasmid (Table 1 and Figure 1).

**Table 1:** The antimicrobial resistance profile and drug resistance genes of *S*. Thompson 17Sal009, the selected transformant (17Sal009T), and the recipient (*E.coli* J53).

**Isolate**	MIC*(mg/L)			**Antibiotic resistance genes on plasmid**
	**CTX**	**CIP**	**NAL**
17Sal009	128	2	32	*bla*_TEM−1_, *bla*_CMY−2_, *aadA2, aph(3”)-Ib, aph(6)-Id, mphA, floR, qnrS1, qepA8, sul1, sul2, tetA, tetR, dfrA12*
17Sal009T	128	2	32	*bla*_TEM−1_, *bla*_CMY−2_, *aadA2, aph(3”)-Ib, aph(6)-Id, mphA, floR, qnrS1, qepA8, sul1, sul2, tetA, tetR, dfrA12*
*E. coli* J53	<0.5	0.015	0.25	

*CTX, cefotaxime; CIP, ciprofloxacin; NAL, nalidixic acid.

The *S*. Thompson isolate 17Sal009 was predicted to have a total of 74 virulence genes implicated in different mechanisms of virulence and pathogenicity, such as *Salmonella* pathogenicity island (SPI)-1 (*hilA, hilC, hilD, invA, invC, invE, invG, invJ, prgH, sipD, spaO, spaR*, and *spas*), SPI-2 (*ssaC, ssaD, ssaL, ssaN, ssaQ, ssaU, ssaV, sseC*, and *ssrA*), which were existed in human isolate (Table 2).

**Table 2:** Virulence genes predicted to exist in *S*. Thompson 17Sal009 and closely related human isolate.

**VF class**	**Virulence factors**	**Human isolate (ID: SAL_UA1616AA)**	**17Sal009**
Fimbrial adherence determinants	Agf/Csg	*csgA, csgB, csgC, csgD, csgE, csgF, csgG*	*csgG*
	Bcf	*bcfA, bcfB, bcfC, bcfD, bcfE, bcfF, bcfG*	*bcfB, bcfC, bcfD*
	Fim	*fimA, fimC, fimD, fimF, fimH, fimI, fimW, fimY, fimZ*	*fimD, fimH, fimZ*
	Lpf	*lpfA, lpfB, lpfC, lpfD, lpfE*	*lpfC, lpfD*
	Peg	*pegA, pegB, pegC, pegD*	*pegC, pegD*
	Saf	*safB, safC*	*safB, safC*
	Stb	*stbB, stbC, stbD, stbE*	*stbB, stbC, stbD*
	Std	*stdA, stdB, stdC*	*stdB*
	Ste	*steA, steB, steC, steD, steE, steF*	*steB*
	Stf	*stfA, stfC, stfD, stfE, stfF, stfG*	*stfC, stfD*
	Sth	*sthA, sthB, sthC, sthD, sthE*	*sthC, sthE*
	Sti	*stiA, stiB, stiC, stiH*	*stiC, stiH*
	Stj	Undetermined (3 genes)	*-*
		*stjB, stjC*	*stjB*
	Stk	*stkA, stkB, stkC, stkD, stkE, stkF, stkG*	*stkB, stkC, stkG*
Macrophage inducible genes	Mig-14	*mig-14*	*mig-14*
Magnesium uptake	Mg2+ transport	*mgtB, mgtC*	*mgtB*
Nonfimbrial adherence determinants	MisL	*misL*	*misL*
	RatB	*ratB*	*ratB*
	ShdA	*shdA*	*shdA*
	SinH	*sinH*	*sinH*
Regulation	PhoPQ	*phoP, phoQ*	*phoQ*
Secretion system	TTSS (SPI-1 encode)	*hilA, hilC, hilD, iacP, iagB, invA, invB, invC, invE, invF, invG, invH, invI, invJ, orgA, orgB, orgC, prgH, prgI, prgJ, prgK, sicA, sicP, sipD, spaO, spaP, spaQ, spaR, spas, sprB*	*hilA, hilC, hilD, invA, invC, invE, invG, invJ, prgH, sipD, spaO, spaR, spas*
	TTSS (SPI-2 encode)	*ssaC, ssaD, ssaE, ssaG, ssaH, ssaI, ssaJ, ssaK, ssaL, ssaM, ssaN, ssaO, ssaP, ssaQ, ssaR, ssaT, ssaU, ssaV, sscA, sscB, sseB, sseC, sseD, sseE, ssrA, ssrB*	*ssaC, ssaD, ssaL, ssaN, ssaQ, ssaU, ssaV, sseC, ssrA*
	TTSS effectors translocated *via* both systems	*slrP*	*slrP*
	TTSS-1 translocated effectors	*avrA, sipA, sipB, sipC, sopA, sopB/sigD, sopD, sopE2, sptP*	*sipA, sipB, sipC, sopA, sopB/sigD, sopD, sptP*
	TTSS-2 translocated effectors	*pipB, sifA, sifB, sseF, sseG, sseJ, sseL, sspH2*	*pipB, sifA, sifB, sseF, sseG, sseJ, sseL, sspH2*
Immune evasion	LPS glucosylation (*Shigella*)	*gtrA*	
Others	O-antigen (Yersinia)	orf02174	orf00369

### Comparative analysis of plasmid pSal009

pSal009 is a 152,940 bp plasmid, with 194 predicated CDSs and an average GC content of 53.4%. The replicon regions were identified as IncA/C by PlasmidFinder. The plasmid includes the core region (*traFHG* and *traNUWCAVBKLDI*) involved in the conjugative transfer, including plasmid replication, horizontal transfer, and stability and maintenance functions, which defines the plasmid backbone (Figure 1) (Call et al., 2010). Various antimicrobial resistance genes were identified on the plasmid, including *floR* encoding phenicol resistance, *tet*(A) and *tet*(R) for tetracycline resistance, *qnrS1* and *qepA8* for quinolone resistance, *dfrA12* for trimethoprim resistance, *mph*(A) for macrolide resistance, *sul1* and *sul2* for sulfonamide resistance, *aadA2, aph(3”)-Ib*, and *aph(6)-Id* for aminoglycoside resistance, and *bla*_CMY−2_ and *bla*_TEM−1_ for beta-lactam resistance (Figure 1). The plasmid also harbors quaternary ammonium resistance genes (*qacE*Δ*1* and *sugE*). A *mer* gene cluster encoding putative regulatory proteins (MerR, MerD), transporters (MerT, MerP, MerE, and MerF) and the mercuric reductase (MerA, MerB), conferring mercurial resistance were identified on the plasmid. In addition, class 1 integron integrase IntI1 and uncharacterized integrase, and copies of transposases and recombinase family protein were observed.

BLASTn comparison of the entire plasmid sequence to microbial sequences in GenBank indicated that it was closely related to pSH11G0791 (GenBank accession number CP041172) (with 99% nucleotide identity and 100% sequence coverage) from a *S*. Thompson strain isolated from human feces in Shanghai, China in 2011, and p846 (GenBank accession number CP029249) (with 99% nucleotide identity and 100% sequence coverage) from a *S*. Thompson strain isolated from children's feces in China in 2014 (Figure 1). Other plasmids with similar backbone include pCMY2_085072 (with 100% nucleotide identity and 86% sequence coverage) (GenBank accession number CP028804) from a clinical *K. pneumoniae* strain, p205880-Ct1/2 (with nucleotide 99% identity and 75% sequence coverage) (GenBank accession number MF344573) from a clinical *K. pneumoniae* strain, and pSL131_IncA/C-IncX3 (with 100% nucleotide identity and 85% sequence coverage) (GenBank accession number MH105050) from a clinical *S*. Lomita strain (Figure 1). The plasmids pCMY2_085072, p205880-Ct1/2 and pSL131_IncA/C-IncX3 carry *bla*_CMY−2_ but lacking *qnrS1, qepA8*, *bla*_TEM−1_, *mph*(A), part of *mer* genes and several insertion sequences (ISs), such as transposases and recombinase family protein.

### Genetic context of ESCs and FQs resistance genes

**Figure 2:**
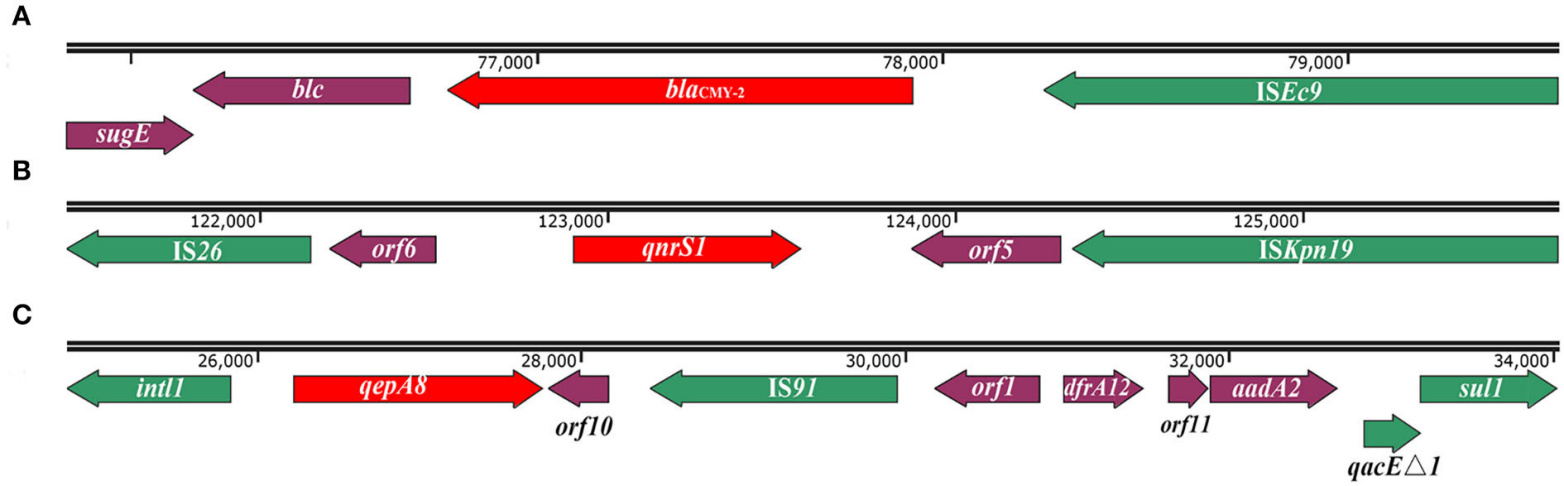
Schematic of genetic context of *bla*_CMY−2_, *qnrS1*, and *qepA8* genes in *S*. Thompson 17Sal009. **(A)** The genetic context of *bla*_CMY−2_ gene. **(B)** The genetic context of the *qnrS1* gene. The *orf5* is predicated to encode recombinase family protein, and *orf6* is predicated to encode transposase. **(C)** The genetic context of the *qepA8* gene. The *orf1* is predicted to encode an uncharacterized site-specific integrase, *orf10* is predicted to encode mechanosensitive ion channel, and *orf11* is predicated to encode DUF1010 domain-containing protein. The arrows indicate open reading frames. Green colors depict mobile genetic elements. Red colors depict *bla*_CMY−2_, *qnrS1*, and *qepA8* genes.

*bla*_CMY−2_ gene is flanked by IS*Ec9* and linked with *sugE* gene and *blc encoding* lipocalin family protein, *sugE*-*blc*-*bla*_CMY−2_-IS*Ec9* (Figure 2A). This structure was found to mainly exist in *E.coli* (blastn resulted 89 sequences of 100% identity matches), and present in small amounts of *S*. Typhimurium, *S*. Anatum, *Salmonella* serovar 1,4,[5],12:i:-, *S*. Heidelberg and *K. pneumoniae* (Supplementary Table S2).

The *qnrS1* gene was located in a composite transposon, IS*26*-*orf6*-*qnrS1*-*orf5*-IS*Kpn19*, in which the *orf6* encoding transposase and the *orf5* encoding recombinase family protein (Figure 2B). In addition to pSH11G0791 and p846, the similar genetic context has also been found in *E.coli, K. pneumoniae, Shigella sonnei*, and *E. fergusonii* (Supplementary Table S3), in which different gene clusters inserted between IS*26* and *orf6* (data not shown). Interestingly, the gene cluster *orf6*-*qnrS1*-*orf5*-IS*Kpn19* has been found in various bacterial species, mainly in *K. pneumoniae* (52 sequences of 100% identity matches), *E.coli* (23 sequences of 100% identity matches), and sporadically in other *Salmonella* serotypes (like *S*. Agona, *S*. Muenster, *Salmonella* serovar 1,4,[5], 12:i:-, *S*. Typhi, *S*. Thompson, *S*. *typhimurium*) or other species including *Shigella sonnei, Serratia liquefaciens, Yokenella regensburgei, Leclercia adecarboxylata*, and *Citrobacter* sp. (Supplementary Table S4).

The *qepA8* gene was located in a class 1 integron with complete structure, *intl1*-*qepA8*-*orf10*-IS*91*-*orf1*-*dfrA12*-*orf11*-*aadA2*-*qacE*Δ*1*-*sul1*, in which the *orf10* encoding mechanosensitive ion channel, *orf1* encoding an uncharacterized site-specific integrase, and *orf11*encoding DUF1010 domain-containing protein (Figure 2C). Except for pSH11G0791 and p846, the same integron has been found in a plasmid in *E.coli* (GenBank accession number CP023959.1) from Urine samples in Canada in 2014 and plasmid in *E.coli* (GenBank accession number MK291500.1) from retail meat from Pakistan in 2018.

### Horizontal transfer of the plasmid

PCR and sequencing results confirmed the successful transfer of the plasmid pSal009 to a plasmid-free recipient *E. coli* J53. Antimicrobial susceptibility testing revealed the acquisition of the plasmid by *E. coli* caused at least a 256-fold increase for cefotaxime, 133-fold increase for ciprofloxacin, and 256-fold increase for nalidixic acid, respectively (Table 1). The conjugation rate was 7.8 × 10^−6^ ± 0.5 transconjugant per recipient cell.

### Phylogenetic analysis

**Figure 3:**
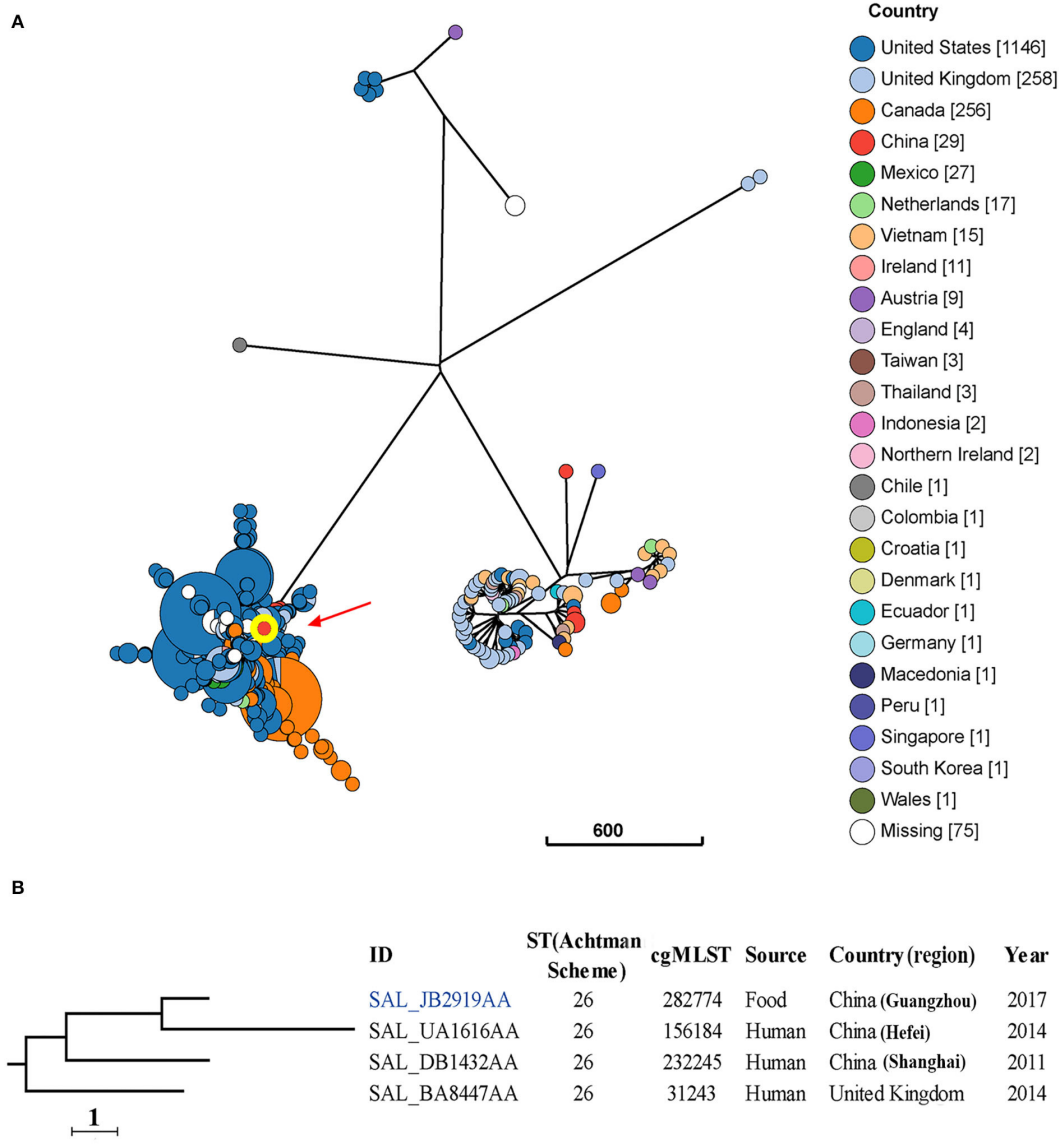
Phylogenetic analysis of 1868 *S*. Thompson isolates from different sources and countries. **(A)** A minimum-spanning tree based on cgMLST analysis. The position of isolate 17Sal009 is indicated by red arrow and highlighted circle. Each circle represents a cgMLST group and the size of the circle is proportional to the number of isolates in that group. **(B)** Detailed information of strains in the branch contained 17Sal009. The isolate 17Sal009 is marked blue.

Five major clusters are seen for the 1868 *S*. Thompson isolates from different countries (Figure 3A). These isolates belong to 19 classical MLST types, with the most frequent being ST26 (94.4%) and ST2125 (2.8%) (Supplementary Figure S1).

cgMLST and phylogenetic analysis showed that the isolate 17Sal009 harbored a unique cgST profile (Figure 3B, Supplementary Table S5), and displayed the closest relationship to one human isolate (ID: SAL_UA1616AA) from Hefei, China in 2014 (Figure 3B), and closely related to a human isolate (ID: SAL_BA8447AA) from Shanghai, China in 2011 as well as a human isolate from the United Kingdom in 2014 (ID: SAL_BA8447AA). cgMLST results differentiated the isolate 17Sal009 with the closest related human isolate up to HC5 level (a maximum of 5 cgMLST allelic variations) and with another two close related isolates up to HC10 level (a maximum of 10 cgMLST allelic variations) (Supplementary Table S4). Therefore, it is inferred that isolate 17Sal009 was highly epidemiologically linked with the human isolate (ID: SAL_UA1616AA) from China in 2014 and was possible epidemiological linked with other two human isolates (ID: SAL_BA8447AA, ID: SAL_BA8447AA).

## Discussion

*Salmonella* resistance to clinically critically important antibiotics ESCs and FQs is a major public health concern (Zhou et al., 2019; Elbediwi et al., 2021). To better understand the origin of the ESCs and FQs resistance in *Salmonella* and its potential effects on human health, it is of major importance to uncover the resistance mechanism as well as their transmission route through the food chain. In this study, we characterized the genetic context of ESCs and FQs resistance genes in an MDR *S*. Thompson isolate recovered from RTE pork product in China.

Previously, MDR profiles in *S*. Thompson have been identified in raw meat (Zhou et al., 2019), RTE meat (Wang et al., 2019), poultry (Xu et al., 2020; Elbediwi et al., 2021), and human (Nair et al., 2016), including ESCs and FQs resistance. However, the genetic determinants of ESCs and FQs resistance in *S*. Thompson have only been sporadically reported, and their genetic contexts as well as transferability remain obscure. A *S*. Thompson isolates from poultry has been identified to carry *bla*_CTX−M−15_ and *qnrB* genes in China (Zhou et al., 2019). Several *S*. Thompson isolates from dead chicks in China were reported to contain *bla*_CTX−M−14_, *bla*_DHA−1_, *bla*_SHV−12_, and *qnrB4* genes (Elbediwi et al., 2021), but the co-occurrence patterns of these resistance genes in individual strains were not available. By retrieving the plasmid sequences from the NCBI database, we found two *S*. Thompson isolates co-harbored the *bla*_CMY−2_, *qnrS1*, and *qepA8* genes, and both of them were obtained from human feces. In this study, to the best of our knowledge, we report the first time a *S*. Thompson isolate from RTE pork product harboring plasmid-mediated *bla*_CMY−2_, *qnrS1*, and *qepA8* genes.

The *bla*_CMY−2_, *qnrS1*, and *qepA8* genes were found to be located on an IncA/C plasmid, which was also identified in two *S*. Thompson isolate of human origin in China. The *bla*_CMY−2_-carrying plasmids were most frequently IncA/C-, IncHI2- or IncX-type plasmids, which were readily transferable between *Salmonella* and *E. coli* from food animals and humans (Shahada et al., 2011). The IncA/C plasmid was observed to be transferable to *E. coli* in the current study, suggesting its transmission potential, which may lead to the development of the ESCs and FQs resistance among different bacterial species.

*bla*_CMY−2_ was found to be located in a transposon-like element consisting of *sugE*-*blc*-*bla*_CMY−2_-IS*Ec9*, in which IS*Ec9* was responsible for mobilization of *bla*_CMY−2_ from the chromosome and transfer onto a plasmid (Fang et al., 2018). This unit has been identified as a typical carrier of the *bla*_CMY−2_ gene (Su et al., 2006; Sidjabat et al., 2014; Yassine et al., 2015) and has been found in many bacterial species, suggesting the highly transferability of *bla*_CMY−2_ gene *via* this unit.

PMQR genes have been regarded as contributive factors to the development of only low-level resistance to fluoroquinolones (ciprofloxacin MICs typically 0.125–2 mg/L) in *Salmonella* (Robicsek et al., 2006; Jiang et al., 2014). In this study, 17Sal009 exhibited a ciprofloxacin MIC of 2 mg/L, which might be due to the synergistic effects of efflux pumps *qepA8* and *qnrS1*, as indicated by the results of a previous study (Chen et al., 2019). Moreover, both *qnrS1* and *qepA8* genes are flanked by mobile genetic elements, *orf6*-*qnrS1*-*orf5*-IS*Kpn19* and *intl1*-*qepA8*-*orf10*-IS*91*-*orf1*-*dfrA12*-*orf11*-*aadA2*-*qacE*Δ*1*-*sul1*, which have also been identified in various species, indicating that these mobile structures could readily transfer from one plasmid to another resulting in the progressive development of ciprofloxacin resistance.

In addition, quaternary ammonium resistance genes (*qacE*Δ*1* and *sugE*) were found to be located on the plasmid, which has been reported to be associated with resistance and enhanced fitness in the intensive-farming environment (Aviv et al., 2014, 2016). Plasmids with similar backbone carrying *bla*_CMY−2_ but lacking many resistance genes and mobile genetic elements were identified in various species, suggesting the *bla*_CMY−2_-bearing plasmid in *S*. Thompson was likely recombined by ISs and integron-mediated recombination activities.

Phylogenomic analysis showed that 17Sal009 was highly epidemiologically linked with a human isolate, suggesting that *S*. Thompson isolates isolated from RTE pork products and humans might come from the same source. Importantly, 17Sal009 contained most of the virulence genes that existed in the closely related human isolate. These genes have been proven to contribute to the strong pathogenicity of *S*. Thompson isolates (Elbediwi et al., 2021; Zhang et al., 2022), suggesting the potential high pathogenicity of 17Sal009 in humans.

The identification of transferable IncA/C plasmid carrying ESCs and FQs resistance genes in Chinese *S*. Thompson isolate from RTE pork product represent potential clinical and food safety issues that need to be monitored since they may transmit to human through the food chain and may lead to reduced susceptibility of *Salmonella* to front-line drugs of choice for treating severe *Salmonella* infections.

## Conclusion

To summarize, this study reports for the first time a *S*. Thompson isolate derived from RTE pork product in China containing a transferable plasmid harboring *bla*_CMY−2_, *qnrS1* and *qepA8* genes. These genes are localized in mobile structures. The transfer of the plasmid and the mobile structures may contribute to the dissemination of *bla*_CMY−2_, *qnrS1*, and *qepA8* genes among different bacterial species and accelerate the development of isolates co-resistant to ESCs and FQs, and this warrants continuous investigations.

## Data availability statement

The datasets presented in this study can be found in online repositories. The names of the repository/repositories and accession number(s) can be found in the article/Supplementary material.

## Author contributions

LL performed the experiment and wrote the manuscript. RO revised the manuscript. HM conceptualized and designed the study. ML and JX provided the strains. SP contributed reagents, materials, and analysis tools. All authors contributed to the article and approved the submitted version.

## Funding

This work was supported by National Natural Science Foundation of China (Grant Numbers 31901789 and 32001796), Natural Science Foundation of Guangdong Province (Grant Numbers 2022A1515011685 and 2020A1515010218), Basic Research Project of Guangzhou (Grant Number 202002030630020049), and Jiangsu Innovative and Enterpreneurial Talent Program (Grant Number JSSCBS20211458).

## Conflict of interest

The authors declare that the research was conducted in the absence of any commercial or financial relationships that could be construed as a potential conflict of interest.

## Publisher's note

All claims expressed in this article are solely those of the authors and do not necessarily represent those of their affiliated organizations, or those of the publisher, the editors and the reviewers. Any product that may be evaluated in this article, or claim that may be made by its manufacturer, is not guaranteed or endorsed by the publisher.
