# Supplementary material for: Characterization of extended-spectrum cephalosporins and fluoroquinolone resistance of a Salmonella enterica serovar Thompson isolate from ready-to-eat pork product in China
Source: Front Microbiol. 2022 Sep 15;13:964009. doi: 10.3389/fmicb.2022.964009 (PMC9521377; doi:10.3389/fmicb.2022.964009)

**Supplementary data**

**Table S2.** Sequences with 100% of coverage and 100% identity with module sugE-blc-bla_CMY-2_-ISEc9 according to Blastn alignment using NCBI GenBank database.

| Taxonomy | Number of sequence of 100% identity matches | Number of Organisms |
| --- | --- | --- |
| *Enterobacteriaceae* | 100 | 10 |
| *. Salmonella enterica* | 9 | 5 |
| *.. Salmonella enterica subsp. enterica* | 1 | 5 |
| *... Salmonella enterica subsp. enterica serovar Typhimurium* | 4 | 1 |
| *... Salmonella enterica subsp. enterica serovar Anatum* | 2 | 1 |
| *... Salmonella enterica subsp. enterica serovar 1,4,[5],12:i:-* | 1 | 1 |
| *... Salmonella enterica subsp. enterica serovar Heidelberg* | 1 | 1 |
| *. Escherichia* | 89 | 4 |
| *.. Escherichia coli* | 80 | 4 |
| *... Escherichia coli O157* | 1 | 1 |
| *... Escherichia coli O16:H48* | 7 | 1 |
| *... Escherichia coli O2:H6* | 1 | 1 |
| *. Klebsiella pneumoniae* | 2 | 1 |

**Table S3.** Sequences with 100% of coverage and 100% identity with module IS26-orf6-qnrS1-orf5-ISKpn19 according to Blastn alignment using NCBI GenBank database.

| GenBank Accession | Bacterial species | Location | Source | Country |
| --- | --- | --- | --- | --- |
| [CP041172.1](https://www.ncbi.nlm.nih.gov/nucleotide/CP041172.1?report=genbank&log$=nucltop&blast_rank=1&RID=FP1ZSVYK014) | [*Salmonella* Thompson](https://blast.ncbi.nlm.nih.gov/Blast.cgi#alnHdr_1695492203) | Plasmid | feces | China |
| [CP029249.1](https://www.ncbi.nlm.nih.gov/nucleotide/CP029249.1?report=genbank&log$=nucltop&blast_rank=2&RID=FP1ZSVYK014) | [*Salmonella* Thompson](https://blast.ncbi.nlm.nih.gov/Blast.cgi#alnHdr_1695492203) | Plasmid | homo sapiens  provincial children's hospital | China |
| [AP023232.1](https://www.ncbi.nlm.nih.gov/nucleotide/AP023232.1?report=genbank&log$=nucltop&blast_rank=3&RID=FP1ZSVYK014) | *Escherichia coli* | Plasmid | - | - |
| [AP023221.1](https://www.ncbi.nlm.nih.gov/nucleotide/AP023221.1?report=genbank&log$=nucltop&blast_rank=4&RID=FP1ZSVYK014) | *Escherichia coli* | Plasmid | healthy Japanese residents | Japan |
| [CP054783.1](https://www.ncbi.nlm.nih.gov/nucleotide/CP054783.1?report=genbank&log$=nucltop&blast_rank=5&RID=FP1ZSVYK014) | *Klebsiella pneumoniae* | Plasmid | sputum | China |
| [CP054777.1](https://www.ncbi.nlm.nih.gov/nucleotide/CP054777.1?report=genbank&log$=nucltop&blast_rank=6&RID=FP1ZSVYK014) | *Klebsiella pneumoniae* | Plasmid | bed sheets | China |
| [CP054771.1](https://www.ncbi.nlm.nih.gov/nucleotide/CP054771.1?report=genbank&log$=nucltop&blast_rank=7&RID=FP1ZSVYK014) | *Klebsiella pneumoniae* | Plasmid | sputum | China |
| [CP054747.1](https://www.ncbi.nlm.nih.gov/nucleotide/CP054747.1?report=genbank&log$=nucltop&blast_rank=8&RID=FP1ZSVYK014) | *Klebsiella pneumoniae* | Plasmid | ventilator | China |
| [CP054741.1](https://www.ncbi.nlm.nih.gov/nucleotide/CP054741.1?report=genbank&log$=nucltop&blast_rank=9&RID=FP1ZSVYK014) | *Klebsiella pneumoniae* | Plasmid | monitor panel | China |
| [CP054753.1](https://www.ncbi.nlm.nih.gov/nucleotide/CP054753.1?report=genbank&log$=nucltop&blast_rank=10&RID=FP1ZSVYK014) | *Klebsiella pneumoniae* | Plasmid | bed sheets | China |
| [CP054735.1](https://www.ncbi.nlm.nih.gov/nucleotide/CP054735.1?report=genbank&log$=nucltop&blast_rank=11&RID=FP1ZSVYK014) | *Klebsiella pneumoniae* | Plasmid | sputum | China |
| [CP054729.1](https://www.ncbi.nlm.nih.gov/nucleotide/CP054729.1?report=genbank&log$=nucltop&blast_rank=12&RID=FP1ZSVYK014) | *Klebsiella pneumoniae* | Plasmid | sputum | China |
| [CP054723.1](https://www.ncbi.nlm.nih.gov/nucleotide/CP054723.1?report=genbank&log$=nucltop&blast_rank=13&RID=FP1ZSVYK014) | *Klebsiella pneumoniae* | Chromosomal | telephone of nurse station | China |
| [CP054759.1](https://www.ncbi.nlm.nih.gov/nucleotide/CP054759.1?report=genbank&log$=nucltop&blast_rank=14&RID=FP1ZSVYK014) | *Klebsiella pneumoniae* | Chromosomal | sputum | China |
| [CP054765.1](https://www.ncbi.nlm.nih.gov/nucleotide/CP054765.1?report=genbank&log$=nucltop&blast_rank=15&RID=FP1ZSVYK014) | *Klebsiella pneumoniae* | Plasmid | sputum | China |
| [AP023198.1](https://www.ncbi.nlm.nih.gov/nucleotide/AP023198.1?report=genbank&log$=nucltop&blast_rank=16&RID=FP1ZSVYK014) | *Escherichia coli* | Plasmid | human stool | Japan |
| [AP023191.1](https://www.ncbi.nlm.nih.gov/nucleotide/AP023191.1?report=genbank&log$=nucltop&blast_rank=17&RID=FP1ZSVYK014) | *Escherichia coli* | Plasmid | human scar swab | Japan |
| [CP045525.2](https://www.ncbi.nlm.nih.gov/nucleotide/CP045525.2?report=genbank&log$=nucltop&blast_rank=18&RID=FP1ZSVYK014) | [*Shigella sonnei*](https://blast.ncbi.nlm.nih.gov/Blast.cgi#alnHdr_1847966279) | Plasmid | stool | Switzerland |
| [LC549808.1](https://www.ncbi.nlm.nih.gov/nucleotide/LC549808.1?report=genbank&log$=nucltop&blast_rank=19&RID=FP1ZSVYK014) | *Klebsiella pneumoniae* | Plasmid | homo sapiens | Viet Nam |

**Table S4.** Sequences with 100% of coverage and significant identity with module orf6-qnrS1-orf5-ISKpn19 according to Blastn alignment using NCBI GenBank database.

| GenBank Accession | Bacterial species | Location | Source | Country |
| --- | --- | --- | --- | --- |
| [CP053046.1](https://www.ncbi.nlm.nih.gov/nucleotide/CP053046.1?report=genbank&log$=nucltop&blast_rank=20&RID=FP1ZSVYK014) | [*Escherichiafergusonii*](https://blast.ncbi.nlm.nih.gov/Blast.cgi#alnHdr_1845853153) | Plasmid | gallusfeces | China |
| [CP049174.1](https://www.ncbi.nlm.nih.gov/nucleotide/CP049174.1?report=genbank&log$=nucltop&blast_rank=21&RID=FP1ZSVYK014) | [*Shigella sonnei*](https://blast.ncbi.nlm.nih.gov/Blast.cgi#alnHdr_1847966279) | Plasmid | stool | Switzerland |
| [CP049186.1](https://www.ncbi.nlm.nih.gov/nucleotide/CP049186.1?report=genbank&log$=nucltop&blast_rank=22&RID=FP1ZSVYK014) | [*Shigella sonnei*](https://blast.ncbi.nlm.nih.gov/Blast.cgi#alnHdr_1847966279) | Plasmid | stool | Switzerland |
| [CP049172.1](https://www.ncbi.nlm.nih.gov/nucleotide/CP049172.1?report=genbank&log$=nucltop&blast_rank=23&RID=FP1ZSVYK014) | [*Shigella sonnei*](https://blast.ncbi.nlm.nih.gov/Blast.cgi#alnHdr_1847966279) | Plasmid | homo sapiens | Switzerland |
| [MT090960.1](https://www.ncbi.nlm.nih.gov/nucleotide/MT090960.1?report=genbank&log$=nucltop&blast_rank=24&RID=FP1ZSVYK014) | *Klebsiella pneumoniae* | Plasmid | blood from male | China |
| [MN539018.1](https://www.ncbi.nlm.nih.gov/nucleotide/MN539018.1?report=genbank&log$=nucltop&blast_rank=25&RID=FP1ZSVYK014) | [*Salmonella sp.*](https://blast.ncbi.nlm.nih.gov/Blast.cgi#alnHdr_1840379582) | Plasmid | duck | China |
| [MN539017.1](https://www.ncbi.nlm.nih.gov/nucleotide/MN539017.1?report=genbank&log$=nucltop&blast_rank=26&RID=FP1ZSVYK014) | [*Salmonella sp.*](https://blast.ncbi.nlm.nih.gov/Blast.cgi#alnHdr_1840379582) | Plasmid | chicken | China |
| [CP052366.1](https://www.ncbi.nlm.nih.gov/nucleotide/CP052366.1?report=genbank&log$=nucltop&blast_rank=27&RID=FP1ZSVYK014) | *Klebsiella pneumoniae* | Plasmid | blood | South Korea |
| [CP052137.1](https://www.ncbi.nlm.nih.gov/nucleotide/CP052137.1?report=genbank&log$=nucltop&blast_rank=28&RID=FP1ZSVYK014) | *Klebsiella pneumoniae* | Plasmid | blood | South Korea |
| [CP052570.1](https://www.ncbi.nlm.nih.gov/nucleotide/CP052570.1?report=genbank&log$=nucltop&blast_rank=29&RID=FP1ZSVYK014) | *Klebsiella pneumoniae* | Plasmid | blood | South Korea |
| [CP052547.1](https://www.ncbi.nlm.nih.gov/nucleotide/CP052547.1?report=genbank&log$=nucltop&blast_rank=30&RID=FP1ZSVYK014) | *Klebsiella pneumoniae* | Plasmid | blood | South Korea |
| [CP052545.1](https://www.ncbi.nlm.nih.gov/nucleotide/CP052545.1?report=genbank&log$=nucltop&blast_rank=31&RID=FP1ZSVYK014) | *Klebsiella pneumoniae* | Plasmid | blood | South Korea |
| [CP052534.1](https://www.ncbi.nlm.nih.gov/nucleotide/CP052534.1?report=genbank&log$=nucltop&blast_rank=32&RID=FP1ZSVYK014) | *Klebsiella pneumoniae* | Plasmid | blood | South Korea |
| [CP052504.1](https://www.ncbi.nlm.nih.gov/nucleotide/CP052504.1?report=genbank&log$=nucltop&blast_rank=33&RID=FP1ZSVYK014) | *Klebsiella pneumoniae* | Plasmid | blood | South Korea |
| [CP052521.1](https://www.ncbi.nlm.nih.gov/nucleotide/CP052521.1?report=genbank&log$=nucltop&blast_rank=34&RID=FP1ZSVYK014) | *Klebsiella pneumoniae* | Plasmid | blood | South Korea |
| [CP052450.1](https://www.ncbi.nlm.nih.gov/nucleotide/CP052450.1?report=genbank&log$=nucltop&blast_rank=35&RID=FP1ZSVYK014) | *Klebsiella pneumoniae* | Plasmid | blood | South Korea |
| [CP052376.1](https://www.ncbi.nlm.nih.gov/nucleotide/CP052376.1?report=genbank&log$=nucltop&blast_rank=36&RID=FP1ZSVYK014) | *Klebsiella pneumoniae* | Plasmid | blood | South Korea |
| [CP052380.1](https://www.ncbi.nlm.nih.gov/nucleotide/CP052380.1?report=genbank&log$=nucltop&blast_rank=37&RID=FP1ZSVYK014) | *Klebsiella pneumoniae* | Plasmid | blood | South Korea |
| [CP052523.1](https://www.ncbi.nlm.nih.gov/nucleotide/CP052523.1?report=genbank&log$=nucltop&blast_rank=38&RID=FP1ZSVYK014) | *Klebsiella pneumoniae* | Plasmid | blood | South Korea |
| [CP052333.1](https://www.ncbi.nlm.nih.gov/nucleotide/CP052333.1?report=genbank&log$=nucltop&blast_rank=39&RID=FP1ZSVYK014) | *Klebsiella pneumoniae* | Plasmid | blood | South Korea |
| [CP052296.1](https://www.ncbi.nlm.nih.gov/nucleotide/CP052296.1?report=genbank&log$=nucltop&blast_rank=40&RID=FP1ZSVYK014) | *Klebsiella pneumoniae* | Plasmid | blood | South Korea |
| [CP052266.1](https://www.ncbi.nlm.nih.gov/nucleotide/CP052266.1?report=genbank&log$=nucltop&blast_rank=41&RID=FP1ZSVYK014) | *Klebsiella pneumoniae* | Plasmid | blood | South Korea |
| [CP052302.1](https://www.ncbi.nlm.nih.gov/nucleotide/CP052302.1?report=genbank&log$=nucltop&blast_rank=42&RID=FP1ZSVYK014) | *Klebsiella pneumoniae* | Plasmid | blood | South Korea |
| [CP052151.1](https://www.ncbi.nlm.nih.gov/nucleotide/CP052151.1?report=genbank&log$=nucltop&blast_rank=43&RID=FP1ZSVYK014) | *Klebsiella pneumoniae* | Plasmid | blood | South Korea |
| [CP052173.1](https://www.ncbi.nlm.nih.gov/nucleotide/CP052173.1?report=genbank&log$=nucltop&blast_rank=44&RID=FP1ZSVYK014) | *Klebsiella pneumoniae* | Plasmid | blood | South Korea |
| [CP052279.1](https://www.ncbi.nlm.nih.gov/nucleotide/CP052279.1?report=genbank&log$=nucltop&blast_rank=45&RID=FP1ZSVYK014) | *Klebsiella pneumoniae* | Plasmid | blood | South Korea |
| [CP051431.1](https://www.ncbi.nlm.nih.gov/nucleotide/CP051431.1?report=genbank&log$=nucltop&blast_rank=46&RID=FP1ZSVYK014) | [*Escherichia sp.*](https://blast.ncbi.nlm.nih.gov/Blast.cgi#alnHdr_1833321251) | Plasmid | swine | China |
| [CP048777.1](https://www.ncbi.nlm.nih.gov/nucleotide/CP048777.1?report=genbank&log$=nucltop&blast_rank=47&RID=FP1ZSVYK014) | [*Salmonella*](https://blast.ncbi.nlm.nih.gov/Blast.cgi#alnHdr_1829108130)Agona | Plasmid | Chroicocephalus novaehollandiae (Australian Silver Gull chick) | Australia |
| [CP048776.1](https://www.ncbi.nlm.nih.gov/nucleotide/CP048776.1?report=genbank&log$=nucltop&blast_rank=48&RID=FP1ZSVYK014) | [*Salmonella*](https://blast.ncbi.nlm.nih.gov/Blast.cgi#alnHdr_1829108130)Agona | Plasmid | Chroicocephalus novaehollandiae (Australian Silver Gull chick) | Australia |
| [CP050812.1](https://www.ncbi.nlm.nih.gov/nucleotide/CP050812.1?report=genbank&log$=nucltop&blast_rank=49&RID=FP1ZSVYK014) | [*Yokenella regensburgei*](https://blast.ncbi.nlm.nih.gov/Blast.cgi#alnHdr_1828977203) | Plasmid | sewage | China |
| [MN842294.1](https://www.ncbi.nlm.nih.gov/nucleotide/MN842294.1?report=genbank&log$=nucltop&blast_rank=50&RID=FP1ZSVYK014) | [*Leclercia adecarboxylata*](https://blast.ncbi.nlm.nih.gov/Blast.cgi#alnHdr_1826684170) | Plasmid | Zhujiang | China |
| [CP050380.1](https://www.ncbi.nlm.nih.gov/nucleotide/CP050380.1?report=genbank&log$=nucltop&blast_rank=51&RID=FP1ZSVYK014) | *Klebsiella pneumoniae* | Plasmid | rectal swab | Czech Republic |
| [MN915011.1](https://www.ncbi.nlm.nih.gov/nucleotide/MN915011.1?report=genbank&log$=nucltop&blast_rank=52&RID=FP1ZSVYK014) | *Escherichia coli* | Plasmid | ceacum | China |
| [MN915010.1](https://www.ncbi.nlm.nih.gov/nucleotide/MN915010.1?report=genbank&log$=nucltop&blast_rank=53&RID=FP1ZSVYK014) | *Escherichia coli* | Plasmid | - | China |
| [MN967025.1](https://www.ncbi.nlm.nih.gov/nucleotide/MN967025.1?report=genbank&log$=nucltop&blast_rank=54&RID=FP1ZSVYK014) | *Klebsiella pneumoniae* | Plasmid | abscess | Taiwan |
| [MK312243.1](https://www.ncbi.nlm.nih.gov/nucleotide/MK312243.1?report=genbank&log$=nucltop&blast_rank=55&RID=FP1ZSVYK014) | *Klebsiella pneumoniae* | Plasmid | - | - |
| [CP050283.1](https://www.ncbi.nlm.nih.gov/nucleotide/CP050283.1?report=genbank&log$=nucltop&blast_rank=56&RID=FP1ZSVYK014) | *Klebsiella pneumoniae* | Plasmid | blood | China |
| [CP050169.1](https://www.ncbi.nlm.nih.gov/nucleotide/CP050169.1?report=genbank&log$=nucltop&blast_rank=57&RID=FP1ZSVYK014) | *Klebsiella pneumoniae* | Plasmid | rectal_swab | Hong Kong |
| [MT035876.1](https://www.ncbi.nlm.nih.gov/nucleotide/MT035876.1?report=genbank&log$=nucltop&blast_rank=58&RID=FP1ZSVYK014) | *Klebsiella pneumoniae* | Plasmid | urine | China |
| [CP048785.1](https://www.ncbi.nlm.nih.gov/nucleotide/CP048785.1?report=genbank&log$=nucltop&blast_rank=59&RID=FP1ZSVYK014) | [*Serratia liquefaciens*](https://blast.ncbi.nlm.nih.gov/Blast.cgi#alnHdr_1812661402) | Plasmid | mixed salads | Germany |
| [MN310373.1](https://www.ncbi.nlm.nih.gov/nucleotide/MN310373.1?report=genbank&log$=nucltop&blast_rank=60&RID=FP1ZSVYK014) | *Klebsiella pneumoniae* | Plasmid | - | - |
| [MN657248.1](https://www.ncbi.nlm.nih.gov/nucleotide/MN657248.1?report=genbank&log$=nucltop&blast_rank=61&RID=FP1ZSVYK014) | [*Enterobacteriaceae bacterium*](https://blast.ncbi.nlm.nih.gov/Blast.cgi#alnHdr_1806595634) | Plasmid | homo sapiens | Germany |
| [LC511658.1](https://www.ncbi.nlm.nih.gov/nucleotide/LC511658.1?report=genbank&log$=nucltop&blast_rank=62&RID=FP1ZSVYK014) | *Escherichia coli* | Plasmid | human stool | Viet Nam |
| [MN783746.1](https://www.ncbi.nlm.nih.gov/nucleotide/MN783746.1?report=genbank&log$=nucltop&blast_rank=63&RID=FP1ZSVYK014) | *Escherichia coli* | Plasmid | - | - |
| [AP022370.1](https://www.ncbi.nlm.nih.gov/nucleotide/AP022370.1?report=genbank&log$=nucltop&blast_rank=64&RID=FP1ZSVYK014) | *Klebsiella pneumoniae* | Plasmid | human stool | Japan |
| [CP027111.1](https://www.ncbi.nlm.nih.gov/nucleotide/CP027111.1?report=genbank&log$=nucltop&blast_rank=65&RID=FP1ZSVYK014) | [*Enterobacter hormaechei*](https://blast.ncbi.nlm.nih.gov/Blast.cgi#alnHdr_1802004100) | Chromosome | homo sapiens | USA |
| [CP023571.1](https://www.ncbi.nlm.nih.gov/nucleotide/CP023571.1?report=genbank&log$=nucltop&blast_rank=66&RID=FP1ZSVYK014) | [*Enterobacter hormaechei*](https://blast.ncbi.nlm.nih.gov/Blast.cgi#alnHdr_1802004100) | Plasmid | blood and wound | USA |
| [CP047092.1](https://www.ncbi.nlm.nih.gov/nucleotide/CP047092.1?report=genbank&log$=nucltop&blast_rank=67&RID=FP1ZSVYK014) | [*Salmonella sp.*](https://blast.ncbi.nlm.nih.gov/Blast.cgi#alnHdr_1799616454) | Plasmid | chicken | China |
| [CP047878.1](https://www.ncbi.nlm.nih.gov/nucleotide/CP047878.1?report=genbank&log$=nucltop&blast_rank=68&RID=FP1ZSVYK014) | *Escherichia coli* | Plasmid | feces | China |
| [CP047667.1](https://www.ncbi.nlm.nih.gov/nucleotide/CP047667.1?report=genbank&log$=nucltop&blast_rank=69&RID=FP1ZSVYK014) | *Escherichia coli* | Plasmid | feces | China |
| [CP032243.1](https://www.ncbi.nlm.nih.gov/nucleotide/CP032243.1?report=genbank&log$=nucltop&blast_rank=70&RID=FP1ZSVYK014) | *Klebsiella pneumoniae* | Plasmid | blood | China |
| [CP046430.1](https://www.ncbi.nlm.nih.gov/nucleotide/CP046430.1?report=genbank&log$=nucltop&blast_rank=71&RID=FP1ZSVYK014) | [*Salmonella enterica*](https://blast.ncbi.nlm.nih.gov/Blast.cgi#alnHdr_1782135532) | Plasmid | blood | India |
| [CP046260.1](https://www.ncbi.nlm.nih.gov/nucleotide/CP046260.1?report=genbank&log$=nucltop&blast_rank=72&RID=FP1ZSVYK014) | *Escherichia coli* | Plasmid | pus | China |
| [LC501656.1](https://www.ncbi.nlm.nih.gov/nucleotide/LC501656.1?report=genbank&log$=nucltop&blast_rank=73&RID=FP1ZSVYK014) | *Escherichia coli* | Plasmid | feces | Japan |
| [MK965545.1](https://www.ncbi.nlm.nih.gov/nucleotide/MK965545.1?report=genbank&log$=nucltop&blast_rank=74&RID=FP1ZSVYK014) | *Escherichia coli* | Plasmid | - | Brazil |
| [CP045559.1](https://www.ncbi.nlm.nih.gov/nucleotide/CP045559.1?report=genbank&log$=nucltop&blast_rank=75&RID=FP1ZSVYK014) | [*Citrobacter sp.*](https://blast.ncbi.nlm.nih.gov/Blast.cgi#alnHdr_1771951380) | Plasmid | - | China |
| [CP045038.1](https://www.ncbi.nlm.nih.gov/nucleotide/CP045038.1?report=genbank&log$=nucltop&blast_rank=76&RID=FP1ZSVYK014) | [*Salmonella*](https://blast.ncbi.nlm.nih.gov/Blast.cgi#alnHdr_1767630627) Muenster | Chromosome | - | China |
| [CP044962.1](https://www.ncbi.nlm.nih.gov/nucleotide/CP044962.1?report=genbank&log$=nucltop&blast_rank=77&RID=FP1ZSVYK014) | [*Salmonella enterica*](https://blast.ncbi.nlm.nih.gov/Blast.cgi#alnHdr_1761798125) | Plasmid | homo sapiens | Canada |
| [AP019676.1](https://www.ncbi.nlm.nih.gov/nucleotide/AP019676.1?report=genbank&log$=nucltop&blast_rank=78&RID=FP1ZSVYK014) | *Escherichia coli* | Plasmid | wastewater treatment plant effluent | Japan |
| [MK649829.1](https://www.ncbi.nlm.nih.gov/nucleotide/MK649829.1?report=genbank&log$=nucltop&blast_rank=79&RID=FP1ZSVYK014) | *Klebsiella pneumoniae* | Plasmid | blood | Laos |
| [CP041376.1](https://www.ncbi.nlm.nih.gov/nucleotide/CP041376.1?report=genbank&log$=nucltop&blast_rank=80&RID=FP1ZSVYK014) | *Klebsiella pneumoniae* | Plasmid | urine | China |
| [CP044256.1](https://www.ncbi.nlm.nih.gov/nucleotide/CP044256.1?report=genbank&log$=nucltop&blast_rank=81&RID=FP1ZSVYK014) | [*Salmonella enterica*](https://blast.ncbi.nlm.nih.gov/Blast.cgi#alnHdr_1752933264) | Plasmid | bulk pig ears | USA |
| [CP044029.1](https://www.ncbi.nlm.nih.gov/nucleotide/CP044029.1?report=genbank&log$=nucltop&blast_rank=82&RID=FP1ZSVYK014) | *Klebsiella pneumoniae* | Plasmid | blood | China |
| [CP044008.1](https://www.ncbi.nlm.nih.gov/nucleotide/CP044008.1?report=genbank&log$=nucltop&blast_rank=83&RID=FP1ZSVYK014) | [*Salmonella*](https://blast.ncbi.nlm.nih.gov/Blast.cgi#alnHdr_1750437643)Typhi | Plasmid | - | - |
| [CP043951.1](https://www.ncbi.nlm.nih.gov/nucleotide/CP043951.1?report=genbank&log$=nucltop&blast_rank=84&RID=FP1ZSVYK014) | *Escherichia coli* | Plasmid | chicken | China |
| [CP043737.1](https://www.ncbi.nlm.nih.gov/nucleotide/CP043737.1?report=genbank&log$=nucltop&blast_rank=85&RID=FP1ZSVYK014) | *Escherichia coli* | Plasmid | chicken legs | USA |
| [CP043751.1](https://www.ncbi.nlm.nih.gov/nucleotide/CP043751.1?report=genbank&log$=nucltop&blast_rank=86&RID=FP1ZSVYK014) | *Escherichia coli* | Plasmid | ground turkey | USA |
| [CP043319.1](https://www.ncbi.nlm.nih.gov/nucleotide/CP043319.1?report=genbank&log$=nucltop&blast_rank=87&RID=FP1ZSVYK014) | [*Enterobacter chengduensis*](https://blast.ncbi.nlm.nih.gov/Blast.cgi#alnHdr_1733474226) | Plasmid | blood | China |
| [LC492467.1](https://www.ncbi.nlm.nih.gov/nucleotide/LC492467.1?report=genbank&log$=nucltop&blast_rank=88&RID=FP1ZSVYK014) | *Escherichia coli* | Plasmid | wound swab, dog | India |
| [CP042974.1](https://www.ncbi.nlm.nih.gov/nucleotide/CP042974.1?report=genbank&log$=nucltop&blast_rank=89&RID=FP1ZSVYK014) | *Escherichia coli* | Plasmid | raw milk cheese | Egypt |
| [CP042900.1](https://www.ncbi.nlm.nih.gov/nucleotide/CP042900.1?report=genbank&log$=nucltop&blast_rank=90&RID=FP1ZSVYK014) | *Escherichia coli* | Plasmid | raw milk cheese | Egypt |
| [CP042902.1](https://www.ncbi.nlm.nih.gov/nucleotide/CP042902.1?report=genbank&log$=nucltop&blast_rank=91&RID=FP1ZSVYK014) | *Escherichia coli* | Plasmid | raw milk cheese | Egypt |
| [CP042872.1](https://www.ncbi.nlm.nih.gov/nucleotide/CP042872.1?report=genbank&log$=nucltop&blast_rank=92&RID=FP1ZSVYK014) | *Escherichia coli* | Plasmid | raw milk cheese | Egypt |
| [CP042586.1](https://www.ncbi.nlm.nih.gov/nucleotide/CP042586.1?report=genbank&log$=nucltop&blast_rank=93&RID=FP1ZSVYK014) | *Escherichia coli* | Plasmid | feces | China |
| [CP033957.1](https://www.ncbi.nlm.nih.gov/nucleotide/CP033957.1?report=genbank&log$=nucltop&blast_rank=94&RID=FP1ZSVYK014) | *Klebsiella pneumoniae* | Chromosome | stool | China |
| [LR596814.1](https://www.ncbi.nlm.nih.gov/nucleotide/LR596814.1?report=genbank&log$=nucltop&blast_rank=95&RID=FP1ZSVYK014) | *Klebsiella pneumoniae* | Chromosome | - | - |
| [LR596813.1](https://www.ncbi.nlm.nih.gov/nucleotide/LR596813.1?report=genbank&log$=nucltop&blast_rank=96&RID=FP1ZSVYK014) | *Klebsiella pneumoniae* | Chromosome | - | - |
| [LR596807.1](https://www.ncbi.nlm.nih.gov/nucleotide/LR596807.1?report=genbank&log$=nucltop&blast_rank=97&RID=FP1ZSVYK014) | *Klebsiella pneumoniae* | Plasmid | - | - |
| [CP040728.1](https://www.ncbi.nlm.nih.gov/nucleotide/CP040728.1?report=genbank&log$=nucltop&blast_rank=98&RID=FP1ZSVYK014) | *Klebsiella pneumoniae* | Plasmid | rectal swab | United Kingdom |
| [CP040574.1](https://www.ncbi.nlm.nih.gov/nucleotide/CP040574.1?report=genbank&log$=nucltop&blast_rank=99&RID=FP1ZSVYK014) | [*Salmonella*](https://blast.ncbi.nlm.nih.gov/Blast.cgi#alnHdr_1750437643) Typhi | Plasmid | blood | Denmark |
| [CP040457.1](https://www.ncbi.nlm.nih.gov/nucleotide/CP040457.1?report=genbank&log$=nucltop&blast_rank=100&RID=FP1ZSVYK014) | [*Salmonella*](https://blast.ncbi.nlm.nih.gov/Blast.cgi#alnHdr_1750437643)Typhimurium | Plasmid | homo sapiens | China |

**Supplementary Table S5.** Sequence type (ST) and core genome MLST (cg MLST) profiles of the [*S*.Thompson](#/javascript:;) isolates that were most closely related to17Sal009 isolate in phylogenetic analysis.

| **Barcode** | **ST (Achtman Scheme)** | **Core Genome Sequence Type (cgST)** | **HC0 (indistinguishable)** | **HC**  **2** | **HC**  **5** | **HC**  **10** | **HC20** | **HC50** | **HC**  **100** | **HC**  **200** | **HC**  **400** | **HC**  **900 (ceBG)** | **HC**  **2000 (Super-lineage)** | **HC**  **2600** | **HC**  **2850 (subsp.)** |  |
| --- | --- | --- | --- | --- | --- | --- | --- | --- | --- | --- | --- | --- | --- | --- | --- | --- |
| SAL_JB2919AA (17Sal009) | 26 | 282774 | 282774 | 230929 | 230929 | 156184 | 31243 | 9058 | 119 | 119 | 119 | 119 | 119 | 2 | 2 |  |
| SAL_UA1616AA | 26 | 156184 | 156184 | 156184 | 156184 | 156184 | 31243 | 9058 | 119 | 119 | 119 | 119 | 119 | 2 | 2 |  |
| SAL_DB1432AA | 26 | 232245 | 232245 | 232245 | 232245 | 31243 | 31243 | 9058 | 119 | 119 | 119 | 119 | 119 | 2 | 2 |  |
| SAL_BA8447AA | 26 | 31243 | 31243 | 31243 | 31243 | 31243 | 31243 | 9058 | 119 | 119 | 119 | 119 | 119 | 2 | 2 |  |

HC: Hierarchical clustering

**Supplementary Figure S1.** Distribution of MLST types among 1868 *S.*Thompson strain.


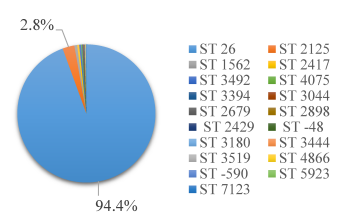

Supplement: Supplementary file 2 [file Data_Sheet_1.docx]
